# Immunoactive Prophylaxis Protocol of Uncomplicated Recurrent Urinary Tract Infections in a Cohort of 1104 Women Treated with Uromune^®^ Vaccine

**DOI:** 10.3390/life14040464

**Published:** 2024-04-02

**Authors:** Cristóbal Ramírez Sevilla, Esther Gómez Lanza, Miguel Puyol Pallàs

**Affiliations:** 1 Fundació Hospital Sant Joan de Déu de Martorell, 08760 Barcelona, Spain; jmpuyol@hmartorell.es; 2 Consorci Sanitari del Maresme, Hospital de Mataró, 08304 Barcelona, Spain; 3 Hospital de Sant Joan Despí Moisés Broggi, 08970 Barcelona, Spain; egomezl@csi.cat

**Keywords:** uncomplicated recurrent UTI, immunoprophylaxis, MV140 vaccine

## Abstract

Background. A prospective, descriptive, and multicenter research that included 1104 women with three or more uncomplicated UTIs following immunoprophylaxis with Uromune^®^ vaccine between 2011 and 2022 is presented. Methods. Objective: to analyze the efficacy of Uromune^®^ and perform a follow-up protocol. Variables: age; bacteria; number of UTIs at baseline and at 3, 6, and 12 months of follow-up; distribution according to age and months of the year; therapy with polybacterial vaccine or autovaccine. Efficacy was defined as 0–2 UTIs during follow-up. Patients were divided into Group 1, with 3–4 UTIs at baseline, and Group 2, with 5 or more. Results. Average age was 72. Escherichia coli represented 64.3% of infections. Overall efficacy was 91.7%, 82.3%, and 57.6% at 3, 6, and 12 months. Efficacy in patients treated with vaccines was 95.8%, 88.4%, and 56.1%, and with autovaccines it was 85.7%, 73.6%, and 60.2%. Results were statistically significant in relation to vaccines (*p* < 0.05). Group 1 represented 65.2% and Group 2 represented 34.8%. Group 1 had an efficacy of 97.7%, 91.1%, and 64.7% and Group 2 had an efficacy of 80.2%, 64.3%, and 40%. Results were statistically significant in Group 1 (*p* < 0.05). Conclusions. Patients at baseline with less than five UTIs will have better result and would benefit from a prophylaxis protocol with Uromune^®^.

## 1. Background

Urinary tract infections have a high incidence and prevalence, which implies frequent use of antibiotics and high healthcare costs, absenteeism from work, impaired quality of life, and increased bacterial resistance due to the continuous intake of antibiotics [1]. Escherichia coli is the most common pathogen for 80% of all UTIs [1]. In the USA, more than 11 million women suffer more than one UTI per year, with a direct cost of USD 659 million [2].

By age 25, approximately 50% of women will have had one UTI, and before menopause 75% will develop two or more UTIs. Throughout their life, 5–8% of women will have had four or more UTIs over a 12-month period, and 25–30% of these will be recurrent [3].

In 2012, the European Association of Urology (EAU) recommended, for the first time, with level 1a scientific evidence, the use of vaccines to prevent recurrent uncomplicated UTI. Additionally, the EAU advises Uro-Vaxom^®^ vaccine in tablets with recommendation grade B, intramuscular Stro-Vac^®^, and parenteral Solco-Urovac^®^ vaccines with recommendation grade C to prevent recurrences [4,5].

In Spain, in October 2010, the Spanish Agency for Medicine and Health Products (AEMPS) approved Uromune^®^ to prevent recurrent UTI. In January 2018, the regulation changed in Spain and now only Uromune^®^ is manufactured as an autovaccine.

In March 2023, based on the results of two retrospective and three prospective studies, the EAU recognized the benefits of Uromune^®^ as a polybacterial sublingual vaccine, which is made from different strains of bacteria to prevent the most common pathogens of UTIs, and stated that Uromune^®^ is better than placebo in reducing the number of recurrent urinary tract infections [6].

Vaccination is considered by the World Health Organization as the most cost-effective strategy to control infectious diseases. Many vaccines have parenteral administration but only a few are for mucosal use. However, the most common infections occur through the mucosa, where secretory immunoglobulin A plays an essential role [7].

Uromune^®^ is applied sublingually and acts on the mucous membranes, activating innate immunity through dendritic cells, natural-killer cells, and macrophages, with an increase of immunoglobulin A secretion that inhibits the adhesion of bacteria to the mucous membranes. Additionally, Uromune^®^ also stimulates humoral immunity through specific antibodies and B and T cells [8].

Over the last decade, different studies have shown good results for Uromune^®^ in relation to efficacy and efficiency, with high tolerance and minimal side effects. Studies of Uromune^®^ have also been published that have shown significant clinical improvement in patients with autoimmune diseases and immunosuppressive treatment; patients with recurrent genital candidiasis; pregnant women; smokers; those with neurogenic bladders, chronic prostatitis, chronic renal disease, renal transplant, and lympho-proliferative disorders; females after trans-obturator tape surgery; and children with urological renal complications [9,10,11,12,13,14].

In 2021, Sánchez-Ramón et al. published a study showing the effect in 55 patients with systemic autoimmune diseases treated with MV130 submucosal vaccine to prevent respiratory infections and MV 140 submucosal vaccine to prevent urinary tract infections. Both types of infections are common in these patients, especially when they follow biological treatment. Forty-one patients completed the vaccination, and after one year of follow-up a significant decrease in UTIs and upper and lower respiratory tract infections was observed [11].

The efficacy and safety of OM-8930 extract against Escherichia coli was investigated in 62 pregnant women with recurrent UTIs during weeks 16 to 28 of pregnancy. The women received one oral extract daily until delivery. UTI recurrences were significantly reduced to 19.4%. Therapy with antibiotics was also reduced by 55.7%. Only one patient had nausea, and another had heartburn. All newborns were healthy with normal Apgar test [12].

A prospective and observational study was conducted from 2018 to 2021 in 49 males with recurrent urinary infections. Of these patients, 53.1% received one round of immunoprophylaxis with autovaccines and 46.9% required two or more rounds. The IPSS score improved by 32% and QoL improved by 54%. Only one patient needed revaccination after three years of follow-up [13].

In 2019, Ramírez et al. published a study on a series of 784 patients with recurrent UTIs. The majority were female, 82.7%, and 17.3% were male. Sublingual prophylaxis was performed with polyvalent vaccine from a collection strain for 3 months. The vaccine was manufactured to cover 25% of each of the four bacteria that most frequently cause UTIs in Spain: *Escherichia coli*, *Klebsiella pneumoniae*, *Proteus vulgaris*, and *Enterococcus faecalis*. Follow-up was carried out at 3 months and 6 months after completion of the treatment. Efficacy was defined as the presence of zero or one UTIs during follow-up. The vaccination achieved an efficacy greater than 71% and 64% at 3 months and 6 months, respectively. Side effects appeared in 1.9% of patients and were mild; no patient stopped the treatment [14].

A study on the effectiveness and efficiency of Uromune^®^ to prevent recurrences of urinary tract infections was recently published in January 2023. In the study, 377 patients, divided into three groups, were compared: 126 received daily prevention with antibiotics for 6 months, 126 received prophylaxis with polyvalent vaccine for 3 months, and 125 received autovaccine for 3 months. Follow-up was carried out at 3 and 6 months. The worst efficacy and highest healthcare cost were achieved in the group treated with antibiotics. The best results corresponded to patients who received immunoprophylaxis with polyvalent vaccine [15].

Other strategies to prevent recurrent UTI, recognized by the 2023 Guidelines of the European Association of Urology, are water intake in premenopausal women greater than 1.5 L/day, with level of evidence 3; vaginal estrogen replacement in postmenopausal women if there is vaginal dryness, with level of evidence 1b; probiotics containing Lactobacillus rhammosus GR-1, Lactobacillus reuteri B-54 and RC-14, Lactobacillus casei Shirota, or Lactobacillus crispatus CTV-05, with level of evidence 1b; use of D-oral mannose, with level of evidence 2; methenamine hippurate tablets, with level of evidence 1b; both continuous low-dose antimicrobial prophylaxis and postcoital antimicrobial prophylaxis, with level of evidence 1b; and cranberry products, with level of evidence 1 and a grade of recommendation of weak [6].

Currently, there is no follow-up protocol for immunoprophylaxis in recurrent UTI.

## 2. Objective

The main objective of this study was to analyze the efficacy of Uromune^®^ vaccine to prevent uncomplicated recurrent UTI and to propose a follow-up protocol.

## 3. Methods

This is a prospective, descriptive, and multicenter research. Three urologists from thtee urban hospitals in Barcelona, Spain, participated: Fundació Hospital de Sant Joan de Déu de Martorell, Hospital de Sant Joan Despí Moisés Broggi, Hospital de Mataró. Hospital Sant Joan Despí Moisés Broggi was the center with the largest reference population, with 500,000 patients. Fundació Hospital Sant Joan de Déu de Martorell had the smallest reference population, approximately 160,000 patients, and the center with intermediate reference was Hospital de Mataró, with 445,000 patients.

UTI was defined as the presence of 100,000 or more germ colony-forming units that colonize and multiply in the urinary system with symptoms.

Urine cultures were analyzed in the laboratory of each of the three participating hospitals, but the criterion for positivity for infection was the same in all three, which was the presence of 100,000 or more bacterial colony-forming units. All patients included in the study had visited the urologist for the first time without prior treatment with vaccines to prevent UTIs.

From January 2011 to May 2023, 1104 consecutive women who had attended the urology department and had been diagnosed with three or more urinary tract infections in the 12 months prior to the first visit were included. All patients received immunoprophylaxis with MV140 sublingually, with a frequency of two puffs every 24 h while fasting over 3 months. To improve the absorption of the vaccine, after application it was advised not to swallow saliva for 1 min, not to eat or drink for 30 min, and not to brush teeth. The treatment was only interrupted in cases of fever of any origin, and once the fever was resolved the vaccine was continued. In the case of therapy with the vaccine, each puff was equivalent to 10^9^ of whole bacteria inactivated by heat in each milliliter, with the same percentage for each of the four most frequent UTI-causing bacteria in Spain: *Escherichia coli*, *Klebsiella pneumoniae*, *Proteus vulgaris*, and *Enterococcus faecalis*. In the case of therapy with the autovaccine, each pulse contained 100% of the bacteria isolated in the urine culture from the sample provided by the patient to the pharmacy office that manufactured the vaccine, and in the case of the growth of two bacteria, 50% of each.

As follow-up, anamnesis and urine culture were performed at 3, 6, and 12 months after the end of treatment. The efficacy of Uromune^®^ was defined as the presence during follow-up of 0, 1, or 2 UTI-positive urine cultures after the end of treatment.

The variables analyzed were age; bacteria; number of UTIs at baseline and at 3, 6, and 12 months of follow-up; distribution according to age groups and months of the year; and therapy with vaccine or autovaccine. Statistical analysis was performed with SPSS version 15.0 (IBM, Chicago, Illinois, USA). To compare proportions, the Chi-square test was used, with Fisher modification when necessary. Comparatives between quantitative variables were performed with Student’s *t*-test.

At baseline, all patients underwent anamnesis as well as abdominal and genital physical examinations, including urine cultures, blood tests, and ultrasounds of the urinary tract with measurement of postvoid volume. In cases of signs of complicated UTI, it was necessary to perform a CT-scan, urethro-cystoscopy, urine cytology, or urodynamics.

Exclusion criteria of the study were patients diagnosed by urodynamics of neurogenic bladder; patients with symptomatic unilateral or bilateral urinary calculi, including renal, ureteral, or bladder; patients with a ureteral pigtail catheter and/or urethral catheter; patients with temporary or permanent nephrostomy; patients with the presence of moderate to severe urinary incontinence, defined as the presence of three or more one-hour pad tests equal to or greater than 50 cc over 24 h; patients with lower urinary tract symptoms (LUTS) in progression, defined as patients with IPSS greater than 20 despite medical treatment with alpha blockers; patients with 5-alpha-reductase inhibitors or both combined; patients with LUTS and cystocele with postvoid volume greater than 100 mL; and patients with urinary diversion such as Bricker, Wallace, cutaneous ureterostomy, ureterosigmoidostomy, or ileal-neobladder.

Patients were divided into two groups according to the number of UTI at baseline. Group 1 represented patients with 3 or 4 UTI, and Group 2 had patients with 5 or more UTI. We compared the results between both groups and performed a follow-up protocol.

All patients received oral and written information about Uromune^®^, that is indication, dosage, side effects, manufacturing, delivery, and urine collection in the case of autovaccine.

Urology outpatient nurse had a fundamental role explaining the information to patients, also avoiding mistakes that may lead to forgetfulness, abandonment or unnecessary second visits. Written informed consent of the patients was recorded.

## 4. Results

The median age of patients was 72 years, with a range of 19–97. In order of frequency, the bacteria isolated were *Escherichia coli*, 64.3%; *Klebsiella pneumoniae*, 24.3%; *Proteus vulgaris*, 5.9%; *Enterococcus faecalis*, 3.5%; *Pseudomona aeruginosa*,1.4%; and *Citrobacter koseri*, 0.5%.

The month of the year when most patients consulted for recurrent UTI was March, with 12.3%, and the month with the fewest was August, with 4.8%. The full distribution by month of the year of recurrent consultations for UTI at baseline was as follows: January, 6.8%; February, 11.3%; March, 12.3%; April, 11%; May, 8.6%; June, 5.3%; July, 7.4%; August, 4.8%; September, 7.2%; October, 10.1%; November, 8.1%; and December, 7.1%.

According to age at baseline, patients were divided into four groups as follows: under 30 years old, 30 to 50 years old, 50 to 70 years old, and over 70 years old. The majority group was over 70 years old. Full distribution by age was under 30 years old, 1.9%; from 30 to 50 years old, 5.4%; from 50 to 70 years old, 26.5%; and over 50 years old, 66.1%.

Autovaccine was administered to 456 patients, 41.3%, and 648 received polyvalent vaccine, 58.7%.

The side effects with Uromune^®^ were mild and represented 1.36% of patients. No patient stopped the treatment. Eight patients presented dry mouth, four gastritis, and three sickness. Side effects were not observed in patients treated with autovaccines.

The number of UTIs at baseline was three UTIs, 40.8%; four UTIs, 24.5%; five UTIs, 19%; six UTIs, 9.8%; seven UTIs, 4.1%; eight UTIs, 1.6%; nine UTIs, 0.2%; and ten UTIs, 0.1%. (Table 1).

Follow-up was registered in 100% of the sample at 3 and 6 months, but at 12 months only 55% follow-up, 611 patients, was recorded.

Three months after immunoprophylaxis, 41.5% had zero UTIs, 30.5% had one UTI, 19.7% had two UTIs, 6.7% had three UTIs, 1.4% had four UTIs, 0.1% had five UTIs, and 0.1% had six UTIs. Efficacy at 3 months was 91.7% (Table 2).

At 6-month follow-up, 26% had zero UTIs, 32.5% had one UTI, 23.8% has two UTIs, 12.9% had three UTIs, 3.9% had four UTIs, 0.5% had five UTIs, 0.3% had six UTIs, and 0.1% had seven UTIs. Efficacy at 6 months was 82.3% (Table 2).

At 12-month follow-up, 9.8% had zero UTIs, 27.5% had one UTI, 20.3% had two UTIs, 25.5% had three UTIs, 13.6% had four UTIs, 2.6% has five UTIs, 0.5% had six UTIs, and 0.2% had seven UTIs. Efficacy at 12 months was 57.6% (Table 2).

Of the patients, 58.7% received vaccines and 41.3% received autovaccines. At 3, 6, and 12 months of follow-up, patients with vaccines had an efficacy of 95.8%, 88.4%, and 56.1%, respectively, and patients treated with autovaccines had 85.7%, 73.6%, and 60.2%, respectively. The analysis between vaccines and autovaccines was statistically significant to vaccines at 3, 6, and 12 months (*p* < 0.05). The overall results with vaccines and autovaccines are presented in Table 3, and the efficacy is presented in Table 4.

Group 1 represented 65.2% of the sample, 720 patients, and Group 2 represented 34.8%, 384 patients. Group 1 had an efficacy of 97.7%, 91.9%, and 64.7% at 3, 6, and 12 months, respectively. In Group 2, the efficacy was 88.2%, 64.3%, and 40% at 3, 6, and 12 months, respectively. The analysis between both groups was statistically significant for Group 1 (*p* < 0.05). The overall results for Groups 1 and 2 are presented in Table 5, and efficacy is presented in Table 6.

**Figure 1:**
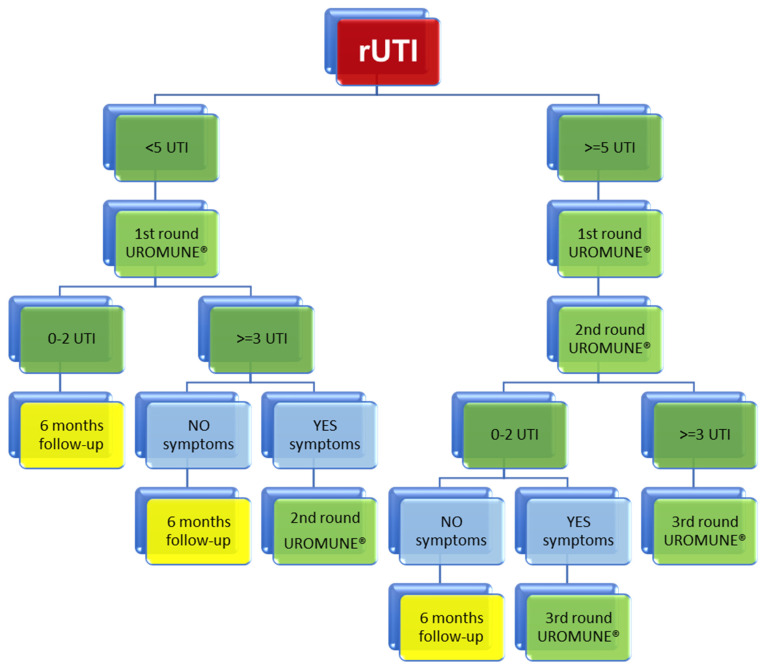
Immunoactive prophylaxis protocol with Uromune^®^ in uncomplicated recurrent UTI.

The results with Uromune^®^ in a large sample of patients and with long follow-up confirmed that patients who initially have less than five UTIs will have a better result than the others, and this is the reason for proposing a new protocol in the immunoactive prophylaxis of uncomplicated recurrent UTIs (Figure 1).

Patients with less than five UTIs at baseline will receive the first round of the vaccine. In the first visit to the urologist after finishing vaccination, if patients present zero to two UTIs, clinical follow-up and urine culture at 6 months will be the best option, but when patients present three or more UTIs without symptoms, follow-up at 6 months will be mandatory, and if patients have three or more UTIs with symptoms, a second round of vaccine will be necessary.

Patients with five or more UTIs at baseline will initially follow two rounds of vaccine. If the results are zero to two UTIs and the absence of symptoms, clinical follow-up with urine culture at 6 months will be the best option. When patients present zero to two UTIs with symptoms, patients have to re-vaccinate on the third round. Finally, if patients have three or more UTIs after the second round, revaccination on the third round is recommended.

## 5. Discussion

In 2015, Lorenzo Gómez et al. published a sample of 360 patients treated with Uromune^®^. The rate of 0–1 UTIs 3 months after vaccination in 90.7% of patients was much greater than the 72% of our study, probably due to the difference in sample size: 360 compared to 1104 [16].

In 2020, Carrión et al. found 0–1 UTIs in 74.4%, 68.1%, and 52.4% of patients at 3, 6, and 12 months, respectively. In contrast, in our study the results were much lower, at 43.5%, 32.2%, and 28.7%. The reason for this was also the difference in sample size: 166 compared to 1104. In any case, the objective of our study was not to reach 0–1 UTIs after vaccination, but to consider that the treatment was effective when it presented 0–2 UTIs [2].

In 2018, Yang et al. published in the *British Journal of Urology International* a study with 75 women diagnosed with recurrent UTI who followed prophylaxis with Uromune^®^. The rate of recurrences was 22% at 12 months of follow-up. In our work, with a sample that is eight-times larger, only 9.8% did not present any urinary infection throughout the 12 months of follow-up. To improve the results on the prevention of recurrent UTI, a re-vaccination protocol could be the best option [17].

Recently, in December 2023, a multicenter study carried out in Australia regarding the use of the sublingual polyvalent MV140 vaccine to prevent recurrent UTIs in people over 65 years was published in the *British Journal of Urology International*. Patients from inpatient and outpatient clinics, all residents of Sydney, were included and randomly divided into two groups: those who received the MV140 vaccine and those who received placebo. Treatment lasted 3 months in both groups, and the planned follow-up was 12 months after finishing prevention. Voiding diary, urine culture, uroflowmetry, and measurement of postvoid residual urine were performed. Additionally, visits to the urologist, emergency attended visits, use of antibiotics to treat UTI recurrences, quality of life, and adverse effects were recorded during follow-up. They defined as a reason for exclusion from the study the presence of significant postvoid urine volume when it was greater than 300mL. However, in our study, in the selection criteria the presence of postvoid residue greater than 100 mL was considered significant and a reason for exclusion. The approach of this study is very interesting, but there was a possible important bias because the follow-up was carried out every 3 months by telephone, not in person at the urologist’s visit. Telephone calls can cause mistakes when transmitting information, especially in elderly people. A total of 150 patients were included, 75 in each group, from August 2023. This study is still open and the results are pending analysis by the end of 2025, but based on the results of previously published papers the results are expected to be promising regarding the use of the MV140 vaccine to prevent UTIs in terms of efficacy and safety [18].

Between 2016 and 2018, 200 frail elderly patients with recurrent UTIs received prophylaxis with MV140 vaccine or Uromune^®^ autovaccine for 3 months. At 12 months of follow-up, the number of recurrences and quality of life were analyzed with the SF-36 questionnaire. The paper, published by Lorenzo-Gómez et al. in *Vaccine Journal*, concluded that both polybacterial vaccines and autovaccines offer significant benefit in reducing the number of UTIs and improving the quality of life of patients. In particular, autovaccines were more effective, a conclusion that agrees with our research, despite having a sample five-times larger [19].

In the last 10 years, several studies have demonstrated high results of immunoprophylaxis with vaccines in recurrent UTI. Vaccines can offer the best efficacy and lower healthcare costs compared to continued antibiotic therapy, without increased bacterial resistance.

To date, our work is the first published with such a large sample of patients, and the first research that analyzes, with 1104 women, an inclusion period of more than 11 years, and follow-up of up to 12 months, the differences according to patients with zero to four UTIs versus five or more. Patients who debut with more than five UTIs, 34.8%, are the worst responders to vaccination and therefore those who will need revaccination early. This analysis is the reason for performing a follow-up protocol in the immunoprophylaxis of recurrent UTI, designed according to the positive or negative urine culture, and presentation or not of symptoms. We believe that it may be useful to unify criteria for action and to identify those patients who are likely to have a worse evolution in order to improve their quality of life with a scheduled revaccination.

The limitations of our study are detailed as follows. The main limitation of this study is the absence of a comparative group with placebo or antibiotic prophylaxis. A group of patients with prophylaxis with placebo has not been created to compare the results because patients who consult with recurrent UTIs frequently have to wait several days or weeks to obtain an appointment with the urologist, and most patients present significant voiding symptoms that can worsen their quality of life, so it would not be appropriate to offer prophylaxis with placebo. A large group with prophylaxis with antibiotics has not been performed to compare the results with vaccines because the authors have considered vaccines as the first option to prevent recurrent UTI, as supported by the EAU guidelines. Follow-up at 12 months in only 55%, 611 patients, is considered another limitation of the study. Satisfaction and quality of life status have not been monitored with questionnaires in patients who received immunoprophylaxis to prevent recurrent UTIs, which is another limitation of our study.

Our experience is extensive and offers high effectiveness, which made consider how to help in the follow-up of these patients, and how to improve the future of the most refractory and recurrent cases by performing a revaccination protocol. This protocol is based on the evidence from our study and determines that patients who present five or more UTIs in the 12 months prior to the first visit will progress worst and therefore they can benefit from more rounds of revaccination from the beginning.

## 6. Conclusions

Immunoactive prophylaxis with Uromune^®^ offers high efficacy in patients with recurrent UTI.

Patients who debut with five or more UTIs will have a worse evolution than other patients.

The follow-up protocol for prophylaxis with Uromune^®^ in women with uncomplicated recurrent UTIs, according to the number of UTIs at baseline, the result of urine culture during follow-up, and the presence or not of symptoms, can be very useful for improving the quality of life of our patients.

Whenever available, polyvalent vaccines are recommended because they offer better results than autovaccines.

## Figures and Tables

**Table 1:** Number and percentage of UTIs at baseline.

UTI Baseline	%
**3**	40.8
**4**	24.5
**5**	19
**6**	9.8
**7**	4.1
**8**	1.6
**9**	0.2
**10**	0.1

**Table 2:** UTI at 3, 6, and 12 months follow-up.

UTI	3 Months (%)	6 Months (%)	12 Months (%)
**0**	41.5	26	9.8
**1**	30.5	32.5	27.5
**2**	19.7	23.8	20.3
**3**	6.7	12.9	25.5
**4**	1.5	3.9	13.6
**5**	0.1	0.5	2.6
**6**	0.1	0.3	0.5
**7**	-	0.1	0.2

**Table 3:** Overall results in patients treated with vaccines and autovaccines.

UTI	Vaccines			Autovaccines		
	3 Months	6 Months	12 Months	3 Months	6 Months	12 Months
**0**	46.6	29.8	9.7	34.2	20.6	10
**1**	30.4	33.6	30.4	30.7	30.9	22.7
**2**	18.8	25	16	20.8	22.1	27.5
**3**	3.9	10.5	25.1	10.7	10.2	26.2
**4**	0.3	0.8	14.9	3.1	8.3	11.4
**5**	-	0.2	2.9	0.2	1.1	2.2
**6**	-	-	0.8	0.2	0.7	-
**7**	-	0.2	0.3	-	-	-

**Table 4:** Efficacy in patients treated with vaccines and autovaccines.

Efficacy	Vaccines	Autovaccines
**3 Months**	95.8	85.7
**6 Months**	88.4	73.6
**12 Months**	56.1	60.2

**Table 5:** Overall results for Groups 1 and 2.

UTI	Group 1			Group 2		
	3 Months	6 Months	12 Months	3 Months	6 Months	12 Months
**0**	52.5	33.2	11.7	20.8	12.5	5.1
**1**	31.9	37.6	30.5	27.9	22.9	20
**2**	13.3	21.1	22.5	31.5	28.9	14.9
**3**	2.2	6.9	21.3	15.1	24	36
**4**	-	1.1	11.5	4.2	9.1	18.9
**5**	-	-	2.1	0.3	1.6	4
**6**	-	-	0.5	0.3	0.8	0.6
**7**	-	-	-	-	0.3	0.6

**Table 6:** Efficacy in Group 1 compared to Group 2.

EFFICACY	Group 1 (<5 UTI)	Group 2 (≥5 UTI)
**3 Months**	98.2%	87.6%
**6 Months**	92.5%	69.4%
**12 Months**	62.1%	40.2%
